# Ten simple rules for training yourself in an emerging field

**DOI:** 10.1371/journal.pcbi.1009440

**Published:** 2021-10-28

**Authors:** Whitney M. Woelmer, L. M. Bradley, Lisa T. Haber, David H. Klinges, Abigail S. L. Lewis, Elizabeth J. Mohr, Christa L. Torrens, Kathryn I. Wheeler, Alyssa M. Willson

**Affiliations:** 1 Department of Biological Sciences, Virginia Tech, Blacksburg, Virginia, United States of America; 2 Department of Biology, Emory University, Atlanta, Georgia, United States of America; 3 Integrative Life Sciences, Virginia Commonwealth University, Richmond, Virginia, United States of America; 4 School of Natural Resources and Environment, University of Florida, Gainesville, Florida, United States of America; 5 Department of Land Resources and Environmental Sciences, Montana State University, Bozeman, Montana, United States of America; 6 Institute of Arctic and Alpine Research, University of Colorado at Boulder, Boulder, Colorado, United States of America; 7 Department of Earth and Environment, Boston University, Boston, Massachusetts, United States of America; 8 Department of Biological Sciences, University of Notre Dame, Notre Dame, Indiana, United States of America; Dassault Systemes BIOVIA, UNITED STATES

## Abstract

The opportunity to participate in and contribute to emerging fields is increasingly prevalent in science. However, simply thinking about stepping outside of your academic silo can leave many students reeling from the uncertainty. Here, we describe 10 simple rules to successfully train yourself in an emerging field, based on our experience as students in the emerging field of ecological forecasting. Our advice begins with setting and revisiting specific goals to achieve your academic and career objectives and includes several useful rules for engaging with and contributing to an emerging field.

## Introduction

**Fig 1:**
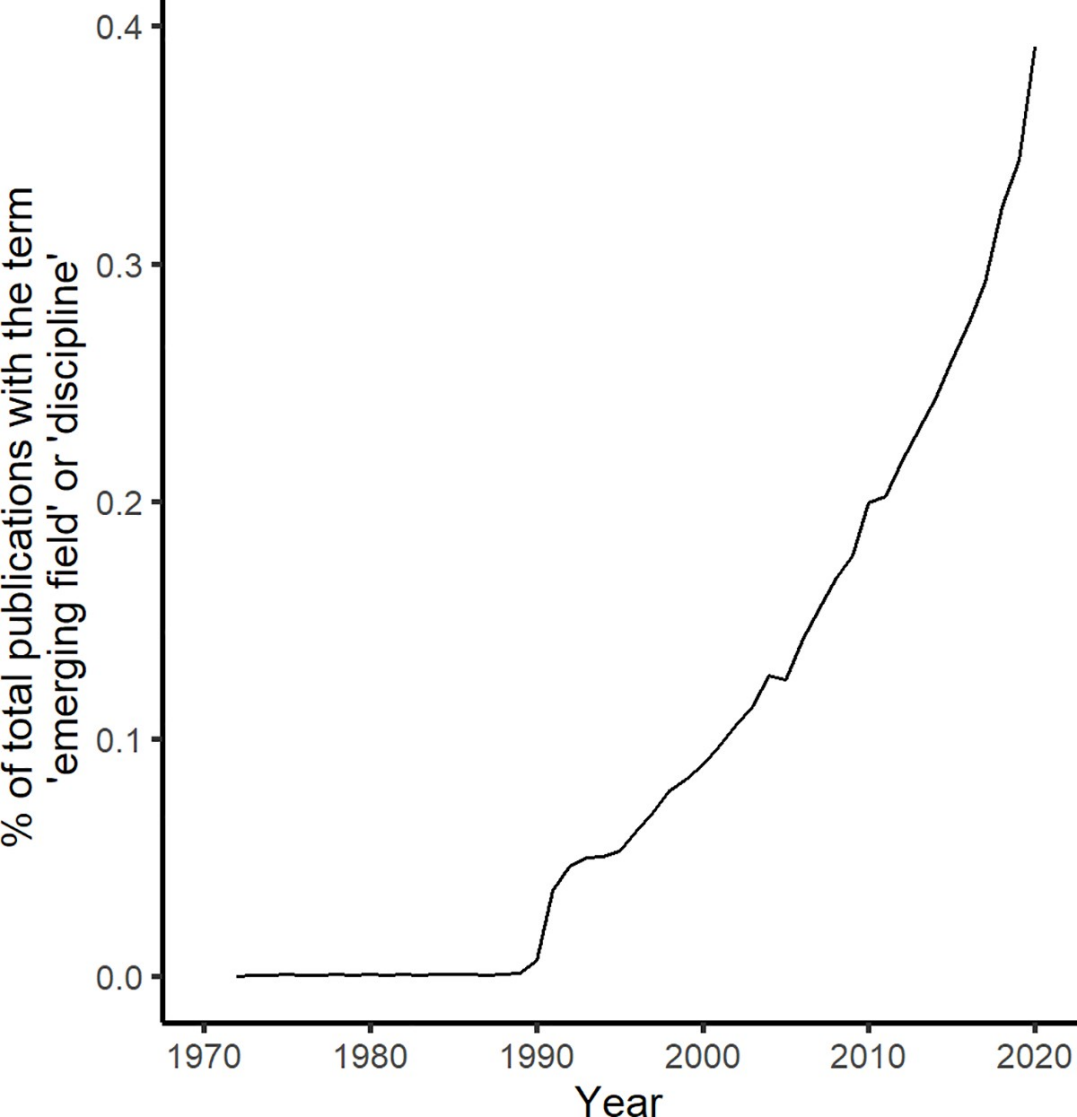
The percent of publications that use the term “emerging field” or “emerging discipline” out of the total publications published in Web of Science from 1970 to 2020.

In an age of increasing data availability [1] and intensified interdisciplinary collaboration [2], emerging disciplines have begun to play a more prominent role in science. Indeed, data from the publication database Web of Science document a steep increase in the percentage of total publications that use the words “emerging discipline” or “emerging field” since 1970 (Fig 1). In light of this growth, the ability to contribute to and participate in emerging fields has become a much needed skill. Emerging fields also offer particularly valuable career development opportunities that students and other early career scientists may be interested in pursuing.

**Fig 2:**
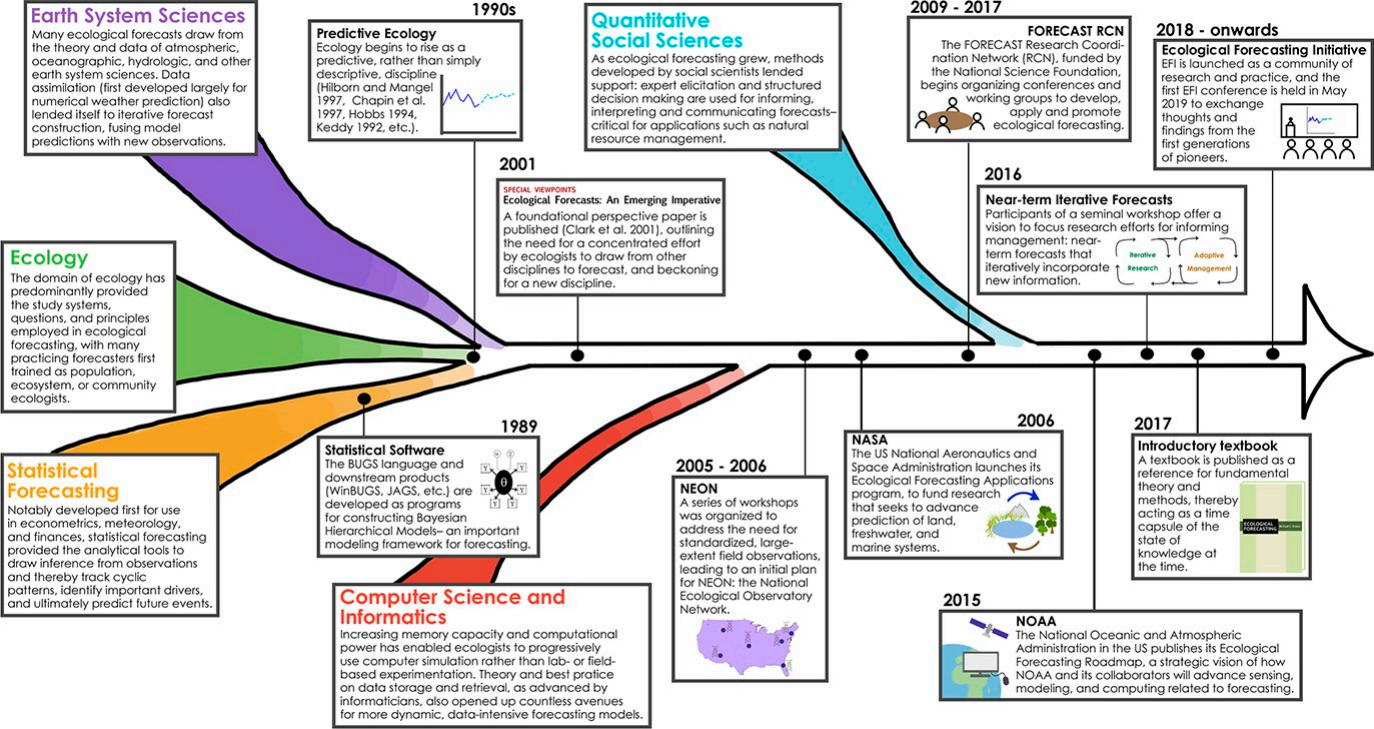
Timeline of the development of ecological forecasting, an emerging field that combines methods, data, and theory from a number of parent fields. Some of the milestones in the field are provided as examples of the accumulation of resources and community building that has occurred thus far.

While there are many definitions for the term “emerging,” here, we define an emerging field as having 2 components. First, it must draw from 2 or more parent fields, which we define as previously established fields of science that are used to inform the development of the emerging field. Second, an emerging field must combine these parent fields to address new or previously unanswerable questions by (1) developing novel methodologies and/or technologies; or (2) applying existing methods or technologies in a novel way. Nanotechnology (e.g., [3], drawn from physics, chemistry, material science, engineering, and biotechnology), social-ecological systems research (e.g., [4], drawn from social psychology, political science, biogeochemistry, and ecology, among others), and ecological forecasting (e.g., [5], drawn from earth system sciences, ecology, statistical forecasting, informatics, and quantitative social sciences; Fig 2) are all examples of emerging fields that have combined techniques or theories from one or more other fields to produce novel and innovative research.

While getting your footing in any discipline requires a high level of self-motivation, entering an emerging field introduces unique hurdles. Where established fields have go-to textbooks and specialized courses as resources, trainees in an emerging field must piece together a wide variety of formal and informal resources from multiple fields. Instead of a single search term to find experts in their fields, a challenge unique to students in an emerging field is that they must look beyond the traditional academic specialties and departments to find those whose skill sets uniquely complement theirs. Nevertheless, students in emerging fields are afforded unique opportunities to pursue leadership roles, contribute to groundbreaking academic pursuits, and shape the future culture of a new, emerging discipline.

Here, we describe 10 simple rules to successfully train yourself in an emerging field. These rules grow out of our own experiences as students in the Ecological Forecasting Initiative (EFI), a growing grassroots network aimed at building and supporting a community of practice around ecological forecasting. As students in an emerging field, we are motivated to use our experience as a guide for other students who may encounter similar struggles in fields that are actively evolving from traditional disciplines. While these 10 simple rules are targeted toward students joining an emerging field, many of the rules may also be applicable to later stage scientists interested in contributing to an emerging field.

### Rule 1: Set goals

Setting goals is one of the most effective ways to ensure a successful project [6], and training yourself in a new area is no exception. Goal setting is especially important in emerging fields where there are less likely to be standard curricula. To maximize the likelihood of achieving your goals, we suggest developing objectives that are specific, measurable, achievable, relevant, and time-oriented (SMART framework, sensu [7]). We recommend the following as a process for setting and meeting your goals:

1. Reflect on why you want to train yourself in the emerging field. Are there particular research questions within the emerging field that excite you? Are there particular career opportunities you wish to work toward?
2. List specific skills and areas of knowledge you need to answer your research questions, land your dream job, etc. In our collective experience, this list is likely to include topics within each parent field, although you may find that your list emphasizes some parent fields more than others.
3. Organize your list of skills and knowledge according to the order in which you intend to learn them. In doing so, it can be helpful to self-evaluate your own current expertise, to determine which skills or which pieces of knowledge are prerequisites for others, and to decide which are your top priorities.
4. Identify your training options (see Rules 2 and 4 to 6), and evaluate the ability of each to meet your learning objectives (Rule 1, Step 2). When evaluating a training option, consider its level (e.g., beginner, intermediate, and advanced) and the depth and breadth of material covered, and identify if you need to reach out for mentorship for these objectives (Rules 4 to 6 can help here). Use your assessment to choose which specific training opportunities to pursue within the next 3 months and within the next year.
5. Ensure that your list is aligned with the SMART framework by developing metrics for measuring your progress (e.g., completion of a course and demonstrated ability to perform a specific type of analysis) and by developing time frames for each measurable outcome.
6. Periodically reevaluate your goals and skills (Rule 9), and revise your training plan if needed (Rule 1, Steps 2 to 5).

### Rule 2: Leverage existing resources, within and outside the emerging discipline

Learning and conducting research in an emerging discipline do not mean that all aspects of the science you are pursuing are entirely novel; rather, it is likely that your research combines the theory and methods from multiple “parent” disciplines to tackle new questions or approach existing questions in new ways. Therefore, the first step in educating yourself in an emerging discipline is to become familiar with the literature, tools, and theory of the parent fields and adjacent fields contributing conceptual or methodological advances. While using resources and knowledge from outside of one’s field is a common, sound practice across scientific fields, it is critical to do so when in an emerging field, as it is less likely that classical, field-specific resources exist, especially those that connect theory from parent fields.

In addition to traditional learning resources such as textbooks and courses, many emerging fields have online resources available, but it may fall on your shoulders to locate and choose among them. For EFI, this has meant searching lab websites, YouTube, Google, and other platforms for videos, lectures, hands-on activities, resource compendiums, and other resources relevant to the field. Further, enthusiastic and knowledgeable collaborators can often serve as extremely valuable resources themselves [8,9]. We therefore suggest that students identify experts and collaborators within the emerging field (Rule 6) both to assist in the search for and prioritization of resources and to serve as mentors throughout one’s training.

Importantly, once you have identified useful resources, consider compiling them in an accessible location for others to find (Rule 3). For example, we are archiving resources relevant to ecological forecasting on the EFI website (https://ecoforecast.org/). These include explanations of important theory from the parent disciplines (e.g., ecological theory), how to practically apply the concepts to your research (e.g., R tutorials), and how to engage with nonacademic stakeholders (e.g., structured decision-making). We emphasize that this effort is best done collectively (see Rules 5 and 6); when resources are shared among collaborators, the directory benefits from diverse research interests and perspectives. Of course, the nature of an emerging discipline means that you will not be able to find every resource you need, no matter how broadly you read and network. Sometimes, you will need to create your own resources (Rule 3).

### Rule 3: Where resources do not exist, help create them

In emerging fields, the resources that researchers normally turn to—textbooks, classes, and conference sessions—may not yet exist. As a result, creating new educational resources in emerging fields is often a critical contribution in improving access to the field. These resources can range from the development of nontraditional courses (i.e., short courses and workshops), to widely used resources such as textbooks, to software tools that enable application of new methods and approaches. While creating some of these resources may be outside the scope of a graduate student’s timeline, graduate students can often lead workshops or create technical resources and may have the opportunity to assist senior scientists in developing larger-scale resources, such as textbooks or intensive training courses. Given that textbooks and other educational materials are most often used by students, in many ways, early career researchers are highly suited for contributing to such resources and developing resources for their peers [10–12]. Here, we identify a few discipline-specific resources that can serve as launching points for an emerging field, which can then develop into more vetted products down the road.

Synchronous courses and workshops are incredibly useful for learning, but such curricula can take years to develop, especially when the knowledge underlying this content is itself evolving. More casual learning sessions, whether in-person or remote, can provide a semistructured way to build understanding through remote walk-throughs of software, serial journal clubs, and group troubleshooting periods. Student-led workshops and resources have been foundational to EFI’s efforts to increase participation broadly, including the annual student workshop developed by the EFI Student Association (EFISA, https://ecoforecast.org/ecological-forecasting-early-career-annual-meeting/), which in 2021, engaged with over 50 early career researchers and provided numerous student-developed tutorials on code reproducibility, model development, utilization of publicly available resources, and presentation of successful submission to the NEON Forecast Challenge (https://ecoforecast.org/efi-rcn-forecast-challenges/).

While educational resources regarding theoretical principles are critical to enabling training in an emerging field, technical resources can be just as necessary. Learning new modeling methods, data structures, or coding grammar can be tedious, and it is likely that other early adopters of your field face the same struggles. As such, making your code available to others can flatten the learning curve for other newcomers. Even as an early career scientist, you can contribute to or lead the creation of discipline-specific software packages that bundle together useful actions, such as downloading data from a domain repository or standardizing variables to accepted units. Short-form tutorials that walk through a data pipeline of assembly, curation, analysis, and/or visualization can offer a recipe to those who are uncertain where to start. Such tutorials need not be thoroughly polished, either; subproducts, screwups, and raw troubleshooting can be just as useful (if not more so) for others seeking to learn alongside you. Building upon stand-alone tutorials, one can contribute to an online repository of tools geared toward a particular discipline or method. For instance, CRAN’s Task Views (https://cran.r-project.org/web/views/) provides overviews of field-specific R software packages. To serve those in the ecological forecasting community, EFI has taken similar steps to provide overviews of methods and tools common to ecological forecasters with an eye toward reproducible workflows, with leadership often coming from early career and student members of the EFI community (https://ecoforecast.org/efi-task-views/). Finally, by making your resources freely available online, you can also help set a precedent of open science in the emerging field and assist underfunded communities who may not have access to proprietary or pay-to-access materials (see Rule 8).

### Rule 4: Attend workshops and conferences

Science is social—many of us know this intuitively, as some of our best research ideas have originated in casual conversations and coffee breaks [13]. For trainees in an emerging field, it may be difficult to find a research sense of community within one’s home institution or existing network. Conferences provide a great opportunity to foster this sense of community while receiving feedback on your research and meeting colleagues doing similar work. While attending conferences is critical for any graduate training program, in emerging fields, it provides an important opportunity to find the breadth of resources and collaborators needed to advance their training in multiple parent fields. Attending conferences regularly and purposefully affords opportunities to discover research and researchers alike, and these interactions can be jumping off points for collaborations or new ideas. Familiarity with the history and parent fields of your discipline (Rule 7), will help you determine (1) what you need from each parent field; and (2) what major conferences to attend to meet these needs. This deliberate planning will maximize your returns, as you cannot attend every conference! Your emerging field (or one closely related to it) may also be hosting sessions as part of a larger conference: Many emerging fields will get started this way as they initially start gaining momentum and building community.

Conferences and workshops give you an opportunity to develop your own sense of belonging in a field: You can network with other researchers who share your interests. Do not be shy! You have opportunities to learn how others got started in this field and where they see it heading (Rules 7 and 8). As a student in an emerging field, navigating your future path and career can be particularly difficult, since the milestones feel unknown. However, by speaking with established researchers, who collectively have diverse backgrounds, you can refine your own path. Additionally, many conferences have professional development sessions for different careers and skills, which can complement the conversations you initiate with researchers.

### Rule 5: Join (or help create!) a network specific to the emerging field

Joining or helping to create a network specific to the emerging field can provide an efficient means of sharing information, building research networks, and coordinating efforts to move the field forward. In emerging fields, several of these benefits are uniquely important. For example, accessing relevant information and resources is often difficult in emerging fields (Rules 2 and 3) without a network for sharing information. Likewise, finding a suitable mentor or collaborator can be particularly difficult in fields with relatively few active participants (Rule 6), but field-specific networks facilitate the development of these connections. Scientific networks in emerging fields will often be smaller than more established organizations, allowing for uniquely close knit communities and active participation from a greater percentage of the organization. Furthermore, in emerging fields, there are frequent opportunities for the development of foundational theory and methods, and working within a network can help coordinate these efforts to advance the field as a whole.

While students alone are likely unable to develop a formal research network, we encourage you to help facilitate and build student-specific networks and collaborations within your emerging field [14,15], as the benefit of peer education has been well documented for many years [10–12]. Work together with other students getting training in your emerging field, whether that’s through informal meetings, journal clubs, or consistent, dedicated time to troubleshoot issues you may be having together. As you work on developing this network, remember that the network is most likely to thrive if you maintain low barriers to entry and encourage inclusivity ([16]; Rule 8). Finally, think carefully about creating and maintaining communication channels that can be used by participants and especially how to take advantage of online collaboration tools (e.g., Twitter, Google Groups, Slack, and Zoom). Communication is a key goal of these networks and will be critical to their success. Similarly, if you have identified a mentor within your emerging field, helping them to coordinate research activities across the emerging field can be both personally and academically rewarding, as you will continue to make meaningful connections within your field.

As members of the EFISA, we have benefitted from being a part of this community and helping to shape its development. We have found that having both a dedicated student working group and participating in EFI’s topical working groups (e.g., Forecasting Theory and Methods, Social Science applications, Education, etc.) have promoted active participation from a growing membership body (*n* = 324, March 2021). Being involved in this community allows us to have conversations with people who have been leaders in the developing field, strengthening our understanding of the field and allowing us to become active participants in moving the field forward. Likewise, conversations with others who are new to the field have offered both a valuable source of support and help with finding resources.

### Rule 6: Find collaborators to work with and learn from

Through your participation in conferences and in a network specific to the emerging field (Rules 4 and 5), you will likely develop collaborative relationships with other scientists. Working cooperatively boosts publishing productivity (as measured by total publication count [17]) advancing career success for all involved. In emerging fields, collaboration is beneficial in a range of additional ways beyond publication totals alone. For example, emerging fields often draw from several existing parent disciplines (Rule 2), and collaborating with researchers whose primary disciplinary background is different from yours can broaden your viewpoint and enhance your network. Likewise, collaborating with researchers who have been involved in the emerging field longer than you offers mentorship that is particularly valuable considering that few other resources may exist to learn about and advance in the emerging field (see Rule 1).

While there are many benefits to collaborative research, developing new, interdisciplinary collaborations also presents meaningful challenges (see [18–20]). Building collaborative relationships is often a slow process, as it takes time to build trust and learn how to communicate across different disciplinary backgrounds [21]. When conducting interdisciplinary research, it is particularly important to learn the “language” that collaborators are using; different disciplines often use different terms to refer to the same concept or use the same term to refer to different things. For example, we have found that terminology such as “forecast horizon” or “data assimilation” are ambiguous in interdisciplinary collaborations, and “short time scale” means something very different to paleoecologists compared to physiological modelers (decades and hours, respectively). For those entering into collaborations as a means of getting training in an emerging field, it is especially important to carefully consider what you can contribute to the project and relationship in addition to how you can benefit from the collaboration. While collaborative science has numerous benefits, it can also require significant investment of time and resources from participants. Be thoughtful and transparent about how much time you can commit and what your goals are from getting involved in a collaboration. Once you have clarified your personal goals, you will be better able to communicate these to others as you jump in and get involved.

### Rule 7: Pay attention to history

Knowing the particular histories of the scientific disciplines in which we work is an important, although often neglected, component of our development as scientists. Learning about the history of your field allows you to appropriately contextualize and relate theoretical advances in the field through time. A primary approach to learning the history of a discipline is to identify and read foundational papers (e.g., [22]). This practice of deep reading in your discipline is useful for any scientist in training in order to learn the origins of key concepts and discipline-specific methodologies. However, the importance of understanding the intellectual development of parent disciplines is heightened in the context of an emerging field, where novel usage of existing theoretical frameworks and analytical approaches necessitates additional caution and awareness of potential limitations. Indeed, we may well ask ourselves as we read foundational papers how their authors might approach long-standing or imperfectly resolved questions given recent advances in theory and technology.

In addition to contextualizing theoretical development over time, studying the history of parent fields allows you to understand and critique the cultural evolution of your parent disciplines with an eye toward avoiding unintended cultures of exclusion. Science (at least in the western hemisphere) has historically been the domain of a relatively small, elite group of white male researchers [23–25]. While recent high-profile papers have illustrated that demographically underrepresented students in science make innovative contributions to their disciplines at higher rates than their historically included counterparts, these contributions are also more likely to be overlooked or ignored due to persistent bias [26]. As Rule 8 explains, the practitioner in an emerging discipline has a unique opportunity to learn from the past in order to build a more equitable future.

Finally, as a trainee in an emerging discipline, you will benefit from exploring the histories of parent disciplines to make sense of a diverse, interdisciplinary array of terms and definitions. A common vocabulary shared among scientists studying similar systems and questions, but at distinct scales and from diverse perspectives, is highly desired, although often elusive. Within EFI, we are working to develop and publish both a working vocabulary of ecological forecasting terms and a basic technical standard for sharing files and metadata, as a means of increasing interoperability of research efforts in this emerging field. We recommend developing this common vocabulary early and using it widely within the emerging field. Ultimately, looking outside the emerging discipline (Rule 2) and through the lens of history allows us to avoid “reinventing the wheel,” and instead apply previously learned lessons to our novel research area.

### Rule 8: Have a vision for the future

As a student and potential future leader in an emerging field, you have a particular stake in helping to shape its future. Relative to fields with longer histories, emerging fields are less likely to have both a well-defined scientific direction and a well-established set of cultural norms, providing a unique opportunity to help create a vision for the field [15]. Start by reflecting on the overarching research questions that excited you and motivated you to join the emerging field. Next, consider any intermediate questions that need to be addressed along the way. What types of data, infrastructure, and collaborations might you need to answer these questions? Using such considerations to prioritize data collection, infrastructure development, and partnership building efforts will allow you to strategically work toward your vision for the emerging field.

In addition to developing a scientific vision, we encourage new members of emerging fields to develop a vision for the field’s culture. We believe that a commitment to both diversity, equity, and inclusivity (DEI), and open science practices (OSPs) will help any emerging field flourish. Here, we describe how and why you might help your field prioritize DEI and OSPs, drawing upon examples from our experience with EFI.

Representation gaps among women, minorities, and persons with disabilities are persistent in the sciences [23]. Even among academic fields that aim to address inequities, established STEM fields have struggled to correct biases even once they have been acknowledged [27–29], leaving an incredible opportunity for emerging fields to start their course right from the beginning. As scientists and humans, we have much to gain from diversity, including faster paced and more rigorous science [30–31], as well as fulfilling a moral and ethical obligation to have diverse representation in science [16]. However, diversity is infeasible without inclusivity and equity, underscoring the need to foster an inclusive culture and provide resources to those who need them [16,32,33]. As a member of an emerging field, you have the chance to lay the foundation for a diverse, equitable, and inclusive field while it is still growing and developing. Inspired by EFI’s DEI initiatives, we suggest several possible avenues for involvement, although many other avenues exist: (1) create a DEI strategic plan; (2) implement inclusive pedagogy practices into your courses [34]; (3) make your lab culture more inclusive [35–37]; and (4) obtain or collect data to track specific gaps in representation in your emerging field.

Concurrent with the movement toward increased DEI in the sciences is a separate but related movement to make science more “open.” OSPs are designed to increase the transparency, accessibility, and reproducibility of scientific research, thereby enabling transfer of knowledge, facilitating collaboration, and enhancing scientific rigor and progress [31,38–41]. While established fields may be entrenched in incentive structures that make engaging in open science difficult [38,42], the fresh culture of emerging fields provides a unique opportunity to establish a collaborative and open scientific culture early on. We encourage those within emerging fields to help establish an open science “culture” by embracing OSPs and/or building infrastructure for the emerging field that facilitates transparency and collaboration. For example, EFI members have established a network for knowledge and skill exchange, facilitated through regular virtual meetings on specific topics within the field. Further, through the work of a National Science Foundation (NSF) Research Coordination Network grant, EFI leaders, including early career members, have developed a set of standardized methods for formatting and archiving forecast output, as well as tools to facilitate the application of these standards. Such standards facilitate the production, sharing, and comparison of a vast diversity of ecological forecasts built upon a common, open-access data source.

By helping to establish norms that promote DEI and OSPs while a field is still emerging, you can propel the field forward in producing more innovative and rigorous science for years to come.

### Rule 9: Measure your success

Continually reevaluating your goals is critical to determining if you are moving along your intended trajectory. First, we encourage you to take the time to self-reflect on the progress you have made toward your goals. This self-evaluation process improves academic success by increasing self-efficacy and motivation, propelling you further down your learning path (Rule 1) [43,44]. One self-evaluation approach is to write down the tools, methods, and papers you are now familiar with that would have been a challenge to digest at an earlier time. Another approach to measuring progress is to look at your contributions to the emerging discipline in the form of resources, community building, or inclusivity. A helpful guiding principle during this self-evaluation is to ask, have I made entering this discipline easier for those who will come next, and, if so, how? Documenting your success and growth over time will help you to advocate for yourself and pursue a fruitful career in the emerging field (Rule 10).

Importantly, as you work toward your goals and develop skills needed in your emerging discipline, your goals and objectives will evolve naturally (Rule 1). At this time, it may be useful to revisit Rule 1 in order to reevaluate your goals and adjust your plan for achieving them.

### Rule 10: Advocate for yourself

While advocating for yourself is important in any career path, it can be especially important and challenging to those in an emerging field. As an emerging field is inherently new, those outside of it may be less familiar with the skill sets and accomplishments associated with success in the field. Thus, communicating and contextualizing your research and expertise to others, ranging from a hiring or promotion committee to friends and family, often require additional thought. It is important to not only develop your brand and elevator pitch [45], but also to develop your pitch in a way that provides sufficient background to those outside your emerging field. The burden of explaining the importance of your field falls more greatly on you, although learning how to advocate for yourself effectively by highlighting the novelty of the emerging field can make it easier.

When communicating your research, it is important to know your audience. Know your audience in terms of general knowledge [46], but also try to identify or predict any preconceived notions they might have about the emerging field. Have they heard of it, or do you have to define the field itself? Are there biases associated with the emerging field? Are they familiar with skills and techniques that you use? If they are unfamiliar with the field, use your skills and techniques as a way to show others what you do. They might not fully understand your research or the emerging field, but being able to connect your research to real-world applications can broaden the perspective of outsiders to the field. For example, ecological forecasters can easily lean on analogies to weather forecasts as a way to familiarize newcomers with the value of the field’s methods.

Especially early in a field’s lifetime, academic departments and nonacademic jobs are unlikely to be seeking out your specific skill set. This might necessitate stepping outside of your comfort zone by thinking creatively about where your skills might be valuable and applying for positions in your field’s “parent” disciplines. For example, ecological forecasters could be housed in a variety of departments such as ecology, biology, earth science, statistics, geography, natural resource management, and public health departments. We recommend being strategic in how you communicate and represent your experiences (e.g., on your CV) to highlight how your unique skill set fits a given job, department, or field, especially if your emerging field is not yet widely recognized.

While it might take additional effort, an exciting aspect of many emerging fields is that they inherently have a vision for novel future research, demonstrating innovative abilities. Thus, while advocating for yourself in an emerging field may be daunting, the novelty and vision of it can strengthen your impact.

## Conclusions

Developing expertise in an emerging field comes with uncertainties and challenges, but there are also many benefits and unique opportunities. Here, we have highlighted some specific actions to help guide students while learning the ropes of an emerging field, ranging from identifying collaborators to finding the right resources to suit your learning needs. While the challenges along the way can seem overwhelming, we also highlight opportunities that come with joining and helping to carve the future of an emerging discipline. By synthesizing methods and theories from multiple established disciplines, emerging fields can both address novel questions and lead to new scientific discovery and pursuit—creating whole new suites of scientific questions. As scientists in emerging fields, we have the additional opportunity to direct the scientific culture of the emerging field, thereby bolstering the scientific community as a whole. We hope that our experience as students training in the field of ecological forecasting can benefit those who seek to find the resources and people to develop their careers in other emerging fields.

## References

[1] Federer LM, Belter CW, Joubert DJ, Livinski A, Lu YL, Snyders LN, et al. Data sharing in PLOS ONE: An analysis of Data Availability Statements. PLoS ONE. 2018;13(5):1–12. doi: 10.1371/journal.pone.0194768 29719004 PMC5931451

[2] Van Noorden R. Interdisciplinary research by the numbers. Nature. 2015;525:306–7. doi: 10.1038/525306a 26381967

[3] Arora SK, Porter AL, Youtie J, Shapira P. Capturing new developments in an emerging technology: an updated search strategy for identifying nanotechnology research outputs. Scientometrics. 2013:351–70.

[4] Liu J, Mooney H, Hull V, Davis SJ, Gaskell J, Hertel T, et al. Systems integration for global sustainability. Science. 2015;347(6225). doi: 10.1126/science.1258832 25722418

[5] Dietze MC, Fox A, Beck-Johnson LM, Betancourt JL, Hooten MB, Jarnevich CS, et al. Iterative near-term ecological forecasting: Needs, opportunities, and challenges. Proc Natl Acad Sci U S A. 2018;115(7):1424–32. doi: 10.1073/pnas.1710231115 29382745 PMC5816139

[6] Locke EA, Latham GP. A theory of goal setting and task performance. Englewood Cliffs, NJ: Prentice Hall; 1990.

[7] Doran GT. There’s a S.M.A.R.T. way to write management’s goals and objectives. Business Source Premier, Management review. 1981;70(11):35.

[8] Long J, McGinnis R. The effects of the mentor on the academic career. Scientometrics. 1985;7:255–80.

[9] Bozeman B, Corley E. Scientists collaboration strategies: implications for scientific and technical human capital. Res Policy. 2004;33(4):599–616.

[10] Damon W. Peer education: The untapped potential. J Appl Psychol. 1984;5(4):331–43.

[11] Colvin JW, Ashman M. Roles, Risks, and Benefits of Peer Mentoring Relationships in Higher Education. Mentor Tutoring. 2010;18(2):121–34.

[12] Latino J, Unite CM. Providing Academic Support through Peer Education. New Dir Higher Educ. 2012;157:39.

[13] Tam V. 2019. Why scientists should take more coffee breaks. Science. 31 October 2019. Available from: https://www.sciencemag.org/careers/2019/10/why-scientists-should-take-more-coffee-breaks.

[14] Shanmugam AK, Macintyre G, Michaut M, Abeel T. Ten Simple Rules for Starting a Regional Student Group. PLoS Comput Biol. 2013;9(11):e1003340. doi: 10.1371/journal.pcbi.1003340 24278001 PMC3836698

[15] Gaëta BA, Rivas JDL, Horton P, Meysman P, Mulder N, Romano P, et al. Ten simple rules for forming a scientific professional society. PLoS Comput Biol. 2017;13(3):e1005226. doi: 10.1371/journal.pcbi.1005226 28333920 PMC5363797

[16] Abernethy E, Arismendi I, Boegehold I, Colon-Gaud C, Cover M, Larson E, et al. Diverse, equitable, and inclusive scientific societies: progress and opportunities in the Society for Freshwater Science. Freshw Sci. 2020;39(3):363–76.

[17] Lee S, Bozeman B. The Impact of Research Collaboration on Scientific Productivity. Soc Stud Sci. 2005;35 (5):673–702.

[18] Vicens Q, Bourne PE. Ten simple rules for a successful collaboration. PLoS Comput Biol. 2007;3:e44. doi: 10.1371/journal.pcbi.0030044 17397252 PMC1847992

[19] de Grijs R. Ten Simple Rules for Establishing International Research Collaborations. PLoS Comput Biol. 2015;11(10):e1004311. doi: 10.1371/journal.pcbi.1004311 26447799 PMC4598231

[20] Frassl MA, Hamilton DP, Denfeld BA, de Eyto E, Hampton SE, Keller PS, et al. Ten simple rules for collaboratively writing a multi-authored paper. PLoS Comput Biol. 2018;14(11):e1006508. doi: 10.1371/journal.pcbi.1006508 30439938 PMC6237291

[21] Kelly R, Mackay M, Nash KL, Cvitanovic C, Allison EH, Armitage D, et al. Ten tips for developing interdisciplinary socio-ecological researchers. Socio Ecol Pract Res. 2019;1:149–61.

[22] Real LA, Brown JH, editors. Foundations of ecology: classic papers with commentaries. 2012. University of Chicago Press: Chicago, Illinois, USA.

[23] National Science Foundation [Internet]. 2019. National Center for Science and Engineering Statistics Directorate for Social, Behavioral, and Economic Sciences. Women, Minorities, and Persons with Disabilities in Science and Engineering. Available from: https://ncses.nsf.gov/pubs/nsf19304/digest/introduction.

[24] Huang J, Gates AJ, Sinatra R, Barabási AL. Historical comparison of gender inequality in scientific careers across countries and disciplines. Proc Natl Acad Sci U S A. 2020;117(9):4609–16. doi: 10.1073/pnas.1914221117 32071248 PMC7060730

[25] Wood S, Henning JA, Chen L, McKibben T, Smith ML, Weber M, et al. A scientist like me: demographic analysis of biology textbooks reveals both progress and long-term lags. Proc R Soc B. 2020;287:20200877. doi: 10.1098/rspb.2020.0877 32576104 PMC7329037

[26] Hofstra B, Kulkarni VV, Galvez SMN, He B, Jurafsky D, McFarland DA. The diversity–innovation paradox in science. Proc Natl Acad Sci U S A. 2020;117(17):9284–91. doi: 10.1073/pnas.1915378117 32291335 PMC7196824

[27] Duran A, Lopez D. Women from Diverse Backgrounds in the Science, Technology, Engineering, and Math (STEM) Professions: Retention and Career Development. In: Hughes C, editor. Impact of Diversity on Organization and Career Development. Hershey, Pennsylvania: IGI Global; 2015. p. 214–251.

[28] Saw G, Chang CN, Chan HY. Cross-Sectional and Longitudinal Disparities in STem Career Aspirations at the Intersection of Gender, race/ethnicity, and Socioeconomic Status. Educ Researcher. 2018;47(8):525–32.

[29] Cech EA, Waidzunas TJ. Systemic inequalities for LGBTQ professionals in STEM. Sci Adv. 2021;7. doi: 10.1126/sciadv.abe0933 33523910 PMC7810386

[30] Swartz TH, Palermo AGS, Masur SK, Aberg JA. The Science and Value of Diversity: Closing the Gaps in Our Understanding of Inclusion and Diversity. J Infect Dis. 2019;220(Suppl 2):S33–41. doi: 10.1093/infdis/jiz174 31430380 PMC6701939

[31] Murphy MC, Mejia AF, Mejia J, Yan X, Cheryan S, Dasgupta N, et al. Open science, communal culture, and women’s participation in the movement to improve science. Proc Natl Acad Sci U S A. 2020;117(39):24154–64. doi: 10.1073/pnas.1921320117 32929006 PMC7533847

[32] Puritty C, Strickland LR, Alia E, Blonder B, Klein E, Kohl MT, et al. Without inclusion, diversity initiatives may not be enough. Science. 2017;357(6356):1101–2. doi: 10.1126/science.aai9054 28912234

[33] Cardel MI, Dhurandhar E, Yarar-Fisher C, Foster M, Hidalgo B, McClure LA, et al. Turning chutes into ladders for women faculty: A review and roadmap for equity in academia. J Womens Health (Larchmt). 2020;29:721–33. doi: 10.1089/jwh.2019.8027 32043918 PMC7247039

[34] Rose DH, Harbour WS, Johnston CS, Daley SG, Abarbanell L. Universal design for learning in postsecondary education: Reflections on principles and their application. J Postsecond Educ Disabil. 2006;19(2):135–51.

[35] Ahmad AS, Sabat I, Trump-Steele R, King E. Evidence-based strategies for improving diversity and inclusion in undergraduate research labs. Front Psychol. 2019;10:1305. doi: 10.3389/fpsyg.2019.01305 31316412 PMC6611382

[36] Powell K. Tech tools to make research more open and inclusive. Nature. 2020;578:181–2. doi: 10.1038/d41586-020-00216-z 32015517

[37] Levine AG. Inclusivity for all: How to make your research group accessible. Science. 23 January 2020. Available from: https://www.sciencemag.org/features/2020/01/inclusivity-all-how-make-your-research-group-accessible.

[38] Nosek BA, Alter G, Banks GC, Borsboom D, Bowman SD, Breckler SJ, et al. Promoting an open research culture. Science. 2015;348 (6242):1422–5. doi: 10.1126/science.aab2374 26113702 PMC4550299

[39] Lowndes J, Best BD, Scarborough C, Afflerbach JC, Frazier MR, O’Hara CC, et al. Our path to better science in less time using open data science tools. Nat Ecol Evol. 2017;1:0160. doi: 10.1038/s41559-017-0160 28812630

[40] Gallagher RV, Falster DS, Maitner BS, Salguero-Gomez R, Vandvik V, Pearse WD, et al. Open Science principles for accelerating trait-based science across the Tree of Life. Nat Ecol Evol. 2020;4:294–303. doi: 10.1038/s41559-020-1109-6 32066887

[41] Parker TH, Forstmeier W, Koricheva J, Fidler R, Hadfield JD, Chee YE, et al. Transparency in Ecology and Evolution: Real Problems. Real Solutions Trends Ecol Evo. 2016;31(9):711–9. doi: 10.1016/j.tree.2016.07.002 27461041

[42] Allen C, Mehler DMA. Open science challenges, benefits, and tips in early career and beyond. PLoS Biol. 2019;17:12. 10.1371/journal.pbio.3000246 PMC6513108 31042704

[43] Schunk DH. Self-efficacy for reading and writing: Influence of modeling, goal setting, and self-evaluation. Read Writ Q. 2003;19(2):159–72.

[44] Martin B, McNally J, Taggar S. Determining the importance of self-evaluation on the goal-performance effect in goal setting: Primary findings. Can J Behav Sci. 2016;48(2):91–100.

[45] Tregoning JS, McDermott JE. Ten Simple Rules to becoming a principal investigator. PLoS Comput Biol. 2020;16(2):e1007448. doi: 10.1371/journal.pcbi.1007448 32078632 PMC7032694

[46] Sura SA, Smith LL, Ambrose MR, Amorim CEG, Beichman AC, Gomez ACR, et al. Ten simple rules for giving an effective academic job talk. PLoS Comput Biol. 2019;15(7):e1007163. doi: 10.1371/journal.pcbi.1007163 31344032 PMC6657819

